# Bioorthogonally Cross‐Linked Hyaluronan–Laminin Hydrogels for 3D Neuronal Cell Culture and Biofabrication

**DOI:** 10.1002/adhm.202102097

**Published:** 2022-02-20

**Authors:** Michael Jury, Isabelle Matthiesen, Fatemeh Rasti Boroojeni, Saskia L. Ludwig, Livia Civitelli, Thomas E. Winkler, Robert Selegård, Anna Herland, Daniel Aili

**Affiliations:** ^1^ Laboratory of Molecular Materials Division of Biophysics and Bioengineering Department of Physics, Chemistry and Biology Linköping University Linköping 581 83 Sweden; ^2^ Division of Micro and Nanosystems KTH Royal Institute of Technology Stockholm 100 44 Sweden; ^3^ Nuffield Department of Clinical Neurosciences John Radcliffe Hospital West Wing University of Oxford Oxford OX3 9DU UK; ^4^ Institute of Microtechnology Center of Pharmaceutical Engineering Technische Universität Braunschweig Braunschweig 38106 Germany; ^5^ AIMES, Center for Integrated Medical and Engineering Science Department of Neuroscience Karolinska Institute Solna 171 65 Sweden; ^6^ Division of Nanobiotechnology Department of Protein Science, Science for Life Laboratory KTH Royal Institute of Technology Stockholm 17165 Sweden

**Keywords:** 3D bioprinting, 3D cell cultures, hyaluronan, hydrogels, laminin, neural stem cells

## Abstract

Laminins (LNs) are key components in the extracellular matrix of neuronal tissues in the developing brain and neural stem cell niches. LN‐presenting hydrogels can provide a biologically relevant matrix for the 3D culture of neurons toward development of advanced tissue models and cell‐based therapies for the treatment of neurological disorders. Biologically derived hydrogels are rich in fragmented LN and are poorly defined concerning composition, which hampers clinical translation. Engineered hydrogels require elaborate and often cytotoxic chemistries for cross‐linking and LN conjugation and provide limited possibilities to tailor the properties of the materials. Here a modular hydrogel system for neural 3D cell cultures, based on hyaluronan and poly(ethylene glycol), that is cross‐linked and functionalized with human recombinant LN‐521 using bioorthogonal copper‐free click chemistry, is shown. Encapsulated human neuroblastoma cells demonstrate high viability and grow into spheroids. Long‐term neuroepithelial stem cells (lt‐NES) cultured in the hydrogels can undergo spontaneous differentiation to neural fate and demonstrate significantly higher viability than cells cultured without LN. The hydrogels further support the structural integrity of 3D bioprinted structures and maintain high viability of bioprinted and syringe extruded lt‐NES, which can facilitate biofabrication and development of cell‐based therapies.

## Graphical abstract

This work presents an extracellular matrix mimicking hydrogel for 3D culture of neural cell models. Based on hyaluronic acid and poly(ethylene glycol), the hydrogel immobilizes recombinant laminin‐521, associated with neuronal development. The study demonstrates support of neuroblastoma cell viability, spontaneous human neuroepithelial stem cell differentiation, and the protective effect of the hydrogels during bioprinting and syringe needle ejection.

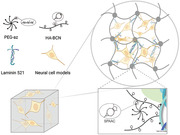

## 1 Introduction

Neurological disorders caused by tumors, degeneration, trauma, infections, congenital or structural defects are combined the second leading cause of death globally.^[^ ^1^, ^2^ ^]^ Access to physiologically relevant human neuronal tissue‐ and disease models is required to improve treatment outcomes and accelerate drug development, which has sparked considerable interest in techniques for generating organoids, organs‐on‐chips and 3D bioprinted constructs with tissue‐ and organ‐like properties.^[^ ^3^, ^4^, ^5^, ^6^ ^]^ New innovative technologies have further facilitated this development for additive manufacturing and advancements in stem cell technologies.^[^ ^7^ ^]^ The latter has also spawned many opportunities for exploring and translating novel therapeutic strategies for neurodegenerative disorders or traumatic injuries.^[^ ^8^, ^9^, ^10^, ^11^ ^]^ Due to the high sensitivity of neural tissues to damage and their minimal regenerative capacity, reparative and regenerative treatment modalities based on stem cell transplantation offer new possibilities to relieve symptoms and restore function after injury or disease.^[^ ^12^, ^13^ ^]^ Both the engineering of functional cellular architectures and the development of cell‐based therapies require well‐defined materials that can mimic the function of the native extracellular matrix (ECM).^[^ ^14^, ^15^, ^16^ ^]^ The ECM offers structural support for cells in all tissues and organs, orchestrates numerous cellular processes, and is critical for cell function and guiding cell behavior and differentiational fate.^[^ ^17^, ^18^ ^]^ The ECM is a dynamic and spatially heterogeneous biomolecular material comprised of glycosaminoglycans (GAGs), proteoglycans, glycoproteins, and fibrous proteins.^[^ ^19^ ^]^ In neural tissues the ECM has a unique composition with large quantities of lecticans and GAGs, such as hyaluronic acid (HA), whilst collagen, vitronectin, fibronectin, and other fibrous proteins are less abundant. ^[^ ^20^, ^21^ ^]^ HA is critical for neuronal development and commonly localized in neural stem cell (NSC) niches.^[^ ^22^, ^23^ ^]^ The developing brain is also rich in laminins (LNs).^[^ ^24^ ^]^ LNs are large heterotrimeric proteins (400–900 kDa), consisting of an *α*, *β*, and *λ*‐chain. LNs are abundant in the basal lamina and are closely associated with neuronal development and known to promote and guide neurite outgrowth^[^ ^25^ ^]^ and to stabilize neuronal synapses.^[^ ^26^ ^]^ More than sixteen different LN trimers have been identified, comprising different combinations of the *α*, *β*, and *λ*‐chains,^[^ ^27^, ^28^ ^]^ and the isoforms are named based on the respective combination of these chains. While several different laminins are relevant in the neurodevelopmental process, ^[^ ^29^ ^]^ we chose to work with the isoform LN‐521 as it has previously been used for in vitro studies of NSC differentiation.^[^ ^30^, ^31^ ^]^

In addition to providing an adequate and biologically relevant microenvironment, ECM mimicking materials developed for biofabrication, and therapeutic applications must be compatible with the required processing conditions, including syringe extrusion, while maintaining high cell viabilities. Whereas biologically derived hydrogels, such as Matrigel, to a certain extent fulfill the requirements for biological relevance, these animal‐derived materials are poorly defined concerning composition and can suffer from large batch‐to‐batch variations that can compromise reproducibility and make clinical translation very challenging.^[^ ^32^, ^33^, ^34^ ^]^ In addition, the limited possibilities to tailor the properties of biologically derived ECM hydrogels make them difficult to adapt to a 3D bioprinting process or to integrate into microfluidic devices for the development of organ‐on‐chips. Engineered ECM mimicking materials are typically designed with the ambition to address these shortcomings. Biopolymers, such as alginate,^[^ ^35^ ^]^ collagen,^[^ ^36^ ^]^ elastin,^[^ ^37^ ^]^ hyaluronic acid,^[^ ^38^ ^]^ and synthetic polymers based on, e.g., poly(ethylene glycol) (PEG),^[^ ^39^ ^]^ poly(vinyl alcohol),^[^ ^40^ ^]^ or poly(2‐hydroxyethyl methacrylate),^[^ ^41^ ^]^ are widely used in the fabrication of ECM mimicking hydrogels. The potential to process the hydrogels and their performance for cell culture is, in addition to composition, highly dependent on polymer cross‐linking chemistry and network topology as well as cross‐linking kinetics and density.^[^ ^42^, ^43^ ^]^ Whereas supramolecular cross‐linking strategies based on molecular self‐assembly, or ion‐coordination are well tolerated by cells and allow for in situ/in vivo gelation, the resulting hydrogels are inherently weak and dynamic, leading to uncontrolled and gradual dissolution over time.^[^ ^44^ ^]^ Covalently cross‐linked hydrogels are typically more robust and can cover a wider stiffness range but often rely on chemistries that can harm cell viability, such as UV‐triggered photo‐polymerization or involve reactions that are difficult to control in a biological context due to cross‐reactivity or poor stability of the functional groups.^[^ ^45^ ^]^ Bioorthogonal strategies, e.g., copper‐free click chemistry, have emerged as attractive options for hydrogel cross‐linking and can facilitate in situ cell encapsulation and biofabrication.^[^ ^38^, ^46^, ^47^, ^48^ ^]^

Because of the critical role of LNs in neural tissue development,^[^ ^49^ ^]^ several different strategies have been developed to incorporate full‐size LN, including LN derived from Engelbreth‐Holm‐Swarm murine sarcoma,^[^ ^50^, ^51^ ^]^ as well as recombinant LN‐411, LN‐111, and LN‐521,^[^ ^52^ ^]^ in engineered ECM mimicking hydrogels to mimic the native 3D microenvironment better. Whereas affinity‐based interactions^[^ ^53^ ^]^ or physical trapping of LN in the hydrogel network^[^ ^52^ ^]^ reduce the risk of interfering with LN structure and function, the LN gradually dissociates from the hydrogels over time. Covalent conjugation can result in more efficient retention of LN in the hydrogels^[^ ^54^, ^55^ ^]^ but can compromise LN function if not carefully optimized. Difficulties in controlling and tuning both cross‐linking kinetics and LN biofunctionalization simultaneously^[^ ^50^, ^51^, ^56^ ^]^ can further complicate the development of generic LN‐presenting hydrogel systems for cell‐based therapeutics and bioinks.

In this work, we have developed an injectable and 3D bioprinting‐compatible modular HA‐based hydrogel system that allows for convenient integration and efficient retention of recombinant LN‐521. LN‐521 is expressed by both neuroepithelial cells and radial glial cells, along with LN‐511 and LN‐111, and are essential for the survival, proliferation, and differentiation of NSCs. We select recombinant LN‐521 since it has been widely used for stem cell expansion and the generation of neural progenitor cells for disease models and stem cell therapies.^[^ ^16^, ^57^, ^58^ ^]^ We cross‐linked the LN‐521 presenting hydrogels by bioorthogonal copper‐free click chemistry, which enabled tuning of material properties and creation of biologically relevant microenvironments evidenced by supported encapsulation and culture of both human neuroblastoma cells (SH‐SY5Y) and human‐induced pluripotent stem cell (hiPSC)‐derived lt‐NES. The strategy for LN conjugation is generic and would also be applicable for other LN isoforms of relevance for neuronal applications, such as LN‐111. Copper‐free click chemistry based on a strain‐promoted azide‐alkyne cycloaddition (SPAAC) is a rapid and reliable method for hydrogel cross‐linking and does not require use of cytotoxic catalysts or UV light.^[^ ^59^ ^]^ Furthermore, the tunable rheological properties of the hydrogels provided a protective effect on the lt‐NES during syringe extrusion in an in vitro model for cell injection therapy. In addition, 3D bioprinting of the cell‐laden hydrogels allowed for the fabrication of structurally well‐defined constructs with high cell viabilities, facilitating further development of advanced tissue and disease models.

## 2 Experimental Section

Detailed methods can be found in the Supporting Information.

### 2.1 Laminin Labeling and Formation of Hydrogel

Hyaluronan–poly(ethylene glycol) (HA:PEG) hybrid hydrogels were prepared by combining bicyclo[6.1.0]nonyne (BCN) modified HA (≈100 kDa) and an 8‐arm PEG with terminal azides ((PEG‐Az)_8_) as previously described.^[^ ^60^, ^61^ ^]^ LN was modified with azide (Az) moieties using linkers of different lengths (LN‐Az and LN‐p‐AZ) and was conjugated to HA‐BCN, after which (PEG‐Az)_8_ was added to form the final hydrogel at 37 °C. The hydrogels were analyzed by rheology and scanning electron microscopy. In addition, the effect of Az‐functionalization on LN retention was measured using fluorescence spectroscopy.

### 2.2 SH‐SY5Y Cell Culture and Differentiation

SH‐SY5Y (ATCC CRL‐2266) is a neuroblastoma cell line originally derived from a metastatic bone tumor and has been shown to express neuronal properties.^[^ ^62^, ^63^ ^]^ Both differentiated and undifferentiated SH‐SY5Y cells were encapsulated in 1% and 2% w/v hydrogels, with and without LN, and cultured for 10 days. Cells were seeded at a density of 2000 cells per µL of hydrogel. Cell viability was assessed using an Alamar blue (AB) assay at 3, 7, and 10 days of culture, and stained for the HA‐receptor cluster of differentiation 44 (CD44) and F‐actin and imaged using confocal fluorescence microscopy.

### 2.3 Encapsulation and Spontaneous 3D Differentiation of lt‐NES

lt‐NES^[^ ^64^ ^]^ C9 were provided by the iPS Core facility (Karolinska Institute) and were previously derived from hiPSC C9.^[^ ^65^ ^]^ 250 000 lt‐NES were encapsulated in 50 µL 1% HA‐based LN functionalized hydrogels, five days after withdrawal of growth factors to induce spontaneous differentiation. lt‐NES were allowed to differentiate spontaneously for an additional 7 days. The results were benchmarked against both Matrigel and 2D cultures on tissue culture plates. The effects of LN on differentiation and viability were investigated using AB at 1, 3, and 7 days of subculture. After 7 days of subculture, the mRNA expression of the stem cell markers Sex determining region Y‐box 2 (SOX2) and Nestin (NES) and the neuronal markers Doublecortin (DCX), Tubulin Beta 3 Class III (TUBB3), and Synapsin‐1 (SYN1) were investigated with qPCR. The hydrogels were further stained for DCX, TUBB3, and F‐actin to visualize the morphology of the cells including process outgrowth by confocal imaging.

### 2.4 Syringe Ejection and 3D Bioprinting

To test whether the hydrogel could serve as a protecting matrix for stem cell therapy applications, lt‐NES were encapsulated in 1% HA:PEG hydrogels and ejected through a 27G syringe needle using a syringe pump in 30 µL hydrogels with 100 000 cells each. Viability was compared to cells suspended in media and ejected under otherwise identical conditions. To optimize the experimental setup, a 5 mg mL^−1^ collagen gel was used initially. After ejection, cell viability was determined both at an immediate stage and after 24 h using a Live/Dead stain. Bioprinting was conducted using a Cellink BioX equipped with a 27G needle at 1 kPa pressure and 25 mm s^−1^ followed by incubation at 37 °C. The bioink was prepared by mixing cells, HA‐BCN and (PEG‐Az)_8_ with or without LN. The bioink was partially cross‐linked for 10 min at room temperature prior to printing. Cell viability was investigated using a Live/Dead assay and imaged using confocal microscopy.

### 2.5 Statistical Analysis

**Figure 1:**
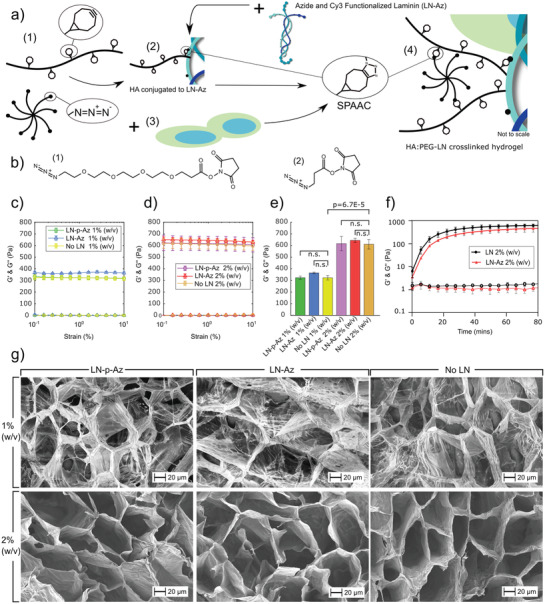
a) Schematic illustration of the modular HA:PEG‐LN hydrogel system: (1) The hydrogel components HA‐BCN and (PEG‐Az)_8_. (2) For conjugation of LN to the hydrogels, Az‐functionalized LN was first conjugated to HA‐BCN prior addition of (PEG‐Az)_8_. (3) For cell encapsulation, cells were suspended in media together with (PEG‐Az)_8_. (4) The (PEG‐Az)_8_ cell suspension was then mixed with HA‐BCN ± LN to generate HA:PEG‐LN. LN was also labeled with Cy3 to determine conjugation efficiency and facilitate visualization of LN distribution. b) LN was functionalized with Az‐terminated amine‐reactive molecules with either (1) a four‐ethylene glycol unit linker (LN‐p‐Az), or (2) a shorter three‐carbon linker (LN‐Az). Oscillatory strain sweeps of HA:PEG‐(LN) hydrogels with concentrations of c) 1% (w/v) and d) 2% (w/v). e) No significant (n.s.; p > 0.05) difference in *G*′ at 1% strain was seen for any of the conditions at the same hydrogel concentration. Statistical analysis is ANOVA with Tukey's HSD. f) Hydrogel gelation kinetics. *N* = 4 for strain sweep and gelation kinetic measurements, where each is a separate hydrogel. Error bars are standard deviation. g) Scanning electron images of hydrogels with and without LN‐(p)‐Az.

Image analysis was performed using ImageJ^[^ ^66^ ^]^ or Fiji.^[^ ^67^ ^]^ Sample sizes (*N*) and *p*‐values (*p*) for all data sets are indicated in the corresponding figure legends. The statistical analysis on AB assayed cell viability (Figure 3) and rheological measurements (**Figure** **1**) was performed using ANOVA followed by a Tukey HSD post hoc test.^[^ ^68^ ^]^ Error bars are mean ± standard deviation and significance was defined as *p* ≤ 0.05. The statistical analysis for the viability of 3D differentiation of lt‐NES (Figure 4), mRNA expression (Figure 5; Figure S6, Supporting Information) and survival of ejected lt‐NES (Figure 6) was performed using Origin Pro (OriginLab, USA). *N* designates individual hydrogel replicates, and *p*‐values were derived using linear mixed models (LMM). Outliers were removed with Grubb's test (Figure 6). For qPCR data (Figure 5; Figure S6, Supporting Information), Ct higher than 35 as well as technical duplicates with values differing by more than 1 were removed. Additional details on calculations can be found in the Supporting Information.

## 3 Results and Discussion

### 3.1 Hydrogel Design

We developed a modular approach based on copper‐free click chemistry to generate HA‐based LN‐521 functionalized hydrogels with tunable stiffness for neural cell encapsulation. HA:PEG hybrid hydrogels were prepared by combining bicyclo[6.1.0]nonyne (BCN) modified HA (≈100 kDa) and an 8‐arm PEG with terminal azide (Az) groups ((PEG‐Az)_8_) as previously described.^[^ ^60^, ^61^ ^]^ The SPAAC reaction between BCN and Az is rapid, allows for efficient and tunable cross‐linking,^[^ ^38^, ^69^ ^]^ and results in optically transparent hydrogels (Figure S1, Supporting Information). To obtain hydrogels with a modulus in the range of neural tissue (*G*′ ≈ 100–1000 Pa)^[^ ^70^, ^71^ ^]^ we prepared the hydrogels at a ratio of BCN to Az of 10:1 and a concentration of 1 and 2% (w/v) of the polymers (Figure 1a), which also preserved a sufficient amount of BCN groups (≈90%) for coupling of LN. For conjugation of LN to the hydrogels, LN‐521 was first modified with Az groups using carbodiimide chemistry.^[^ ^72^ ^]^ The Az groups were coupled to LN using linkers of two lengths to optimize LN conjugation and retention in the hydrogels. The longer linker comprised four ethylene glycol units, and the shorter was based on a three‐carbon linker, referred to as LN‐p‐Az and LN‐Az, respectively (Figure 1b). After purification, LN‐Az/LN‐p‐Az (LN‐(p)‐Az) was combined with HA‐BCN to allow HA‐BCN to bind to LN. We then cross‐linked the constructs by the addition of (PEG‐Az)_8_ (Figure 1a). In the absence of LN, the storage modulus (*G*′) of the hydrogels was ≈350 and 650 Pa for 1% and 2% (w/v) hydrogels, respectively, which is in the desired range for the culture of neurons (Figure 1c,d). Previous works by Saha et al.^[^ ^73^ ^]^ and Banerjee et al.^[^ ^74^ ^]^ have demonstrated that hydrogels in this stiffness range enhance proliferation and differentiation of NSCs compared to when cultured in stiffer gels. The addition of LN‐Az or LN‐p‐Az (0.83 × 10^−6^ m) did not have any significant effect on the stiffness of the hydrogels (Figure 1c–e) nor the gelation kinetics (Figure 1f). The gelation point (*G*′ = *G*″) was reached directly after mixing the components at 37 °C in PBS, and the hydrogels reached close to final stiffness in about 20 min. This time frame is sufficiently fast to prevent cell sedimentation while allowing enough time for handling and bioprinting. In previous work, we have also observed that the gelation kinetics for SPAAC cross‐linking is highly temperature dependent and gelation can be delayed significantly when performed at room temperature and almost completely inhibited at 4 °C, ^[^ ^38^ ^]^ which can further facilitate processing of the hydrogels.

Scanning electron images of freeze‐dried hydrogels did not reveal any substantial differences between the LN and non‐LN‐containing hydrogels (Figure 1g). All hydrogels showed large hexagonal and interconnected pores, 50–100 µm in size. The lower weight percentage hydrogels, 1% (w/v), showed thinner pore walls and a more fibrillar structure than hydrogels prepared at a concentration of 2% (w/v). Pores in this size range can facilitate cell migration and cell–cell contacts and diffusion of oxygen, nutrients, and other critical factors for cell survival, proliferation, and function without a vascular system.^[^ ^75^, ^76^ ^]^ The porous microarchitecture can also influence and promote neurite outgrowth.^[^ ^76^, ^77^ ^]^

### 3.2 Laminin Distribution and Retention

**Figure 2:**
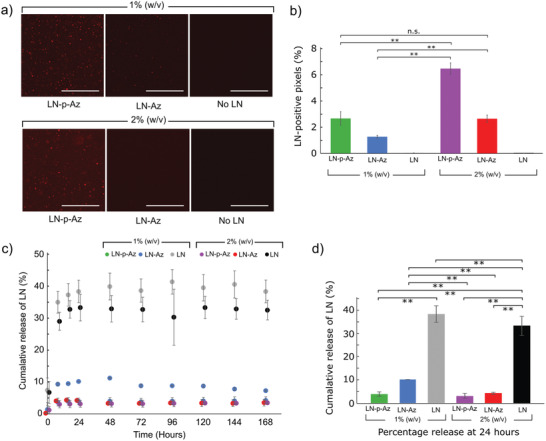
a) Fluorescence images of the Cy3‐labeled LN‐(p)‐Az conjugated in the HA:PEG hydrogels. Scale bars: 100 µm. b) LN‐positive pixels determined from fluorescence images of the hydrogels, *N* = 4 for each condition, n.s. = not significant (*p* > 0.05). c) Cumulative release of unbound LN, LN‐p‐Az, and LN‐Az from hydrogels with 1% and 2% (w/v) for 7 days, *N* = 6. d) Percentage release at 24 h showing statistical significance pairings where ** = *p* < 0.01 using ANOVA followed by Tukey's HSD. All error bars are standard deviation.

To characterize the influence of Az modification and linker length on the retention of LN in the hydrogels, we further labeled the LN with Cy3. Fluorescence images of the hydrogels functionalized with Cy3‐labeled LN‐(p)‐Az show a homogenous distribution of the LN with a small number of visible aggregates (**Figure** **2**a). Based on the relative intensity of LN‐Cy3 from the fluorescence images, we can conclude that about twice as much LN was conjugated to the 2% (w/v) compared to the 1% (w/v) hydrogels and that LN‐p‐Az features more efficient conjugation compared to LN‐Az (Figure 2b). To further determine the effectiveness of the conjugation strategies, we monitored the cumulative release of LN for 7 days (Figure 2c). For the non‐Az functionalized LN, a substantial burst release was seen over the first 24 h, corresponding to about 40% of the incorporated LN (Figure 2d). However, after the initial burst release, limited further release was observed, and a large fraction of the nonconjugated LN was consequently physically trapped in the hydrogel. This is likely due to the high molecular weight of LN (≈850 kDa), resulting in a slow diffusion in the hydrogel polymer network. However, LN modified with Az via both the shorter (LN‐Az) and the longer and more flexible linker (LN‐p‐Az) was found to be substantially more efficiently retained in the hydrogels with a cumulative release of less than 5% for the 2% (w/v) HA:PEG hydrogels, indicating successful conjugation to the HA backbone.

### 3.3 Encapsulation and 3D Culture of SH‐SY5Y Cells

Physical trapping of full‐length recombinant LN‐521 in spider‐silk‐based hydrogels has previously been demonstrated to support expansion of human pluripotent stem cells and subsequent neural differentiation.^[^ ^52^ ^]^ LNs covalently conjugated to oxidized alginate/gelatin hydrogels further facilitate neuronal differentiation and growth of embedded hiPSC‐derived neurospheres.^[^ ^56^ ^]^ LN conjugated to oxidized methyl cellulose‐based hydrogels using a Schiff base reaction was also found to support cell attachment and survival of primary neurons isolated from embryonic day 18 Sasco Sprague‐Dawley rats.^[^ ^50^ ^]^ Encouraged by these findings and the possibilities to efficiently conjugate and retain LN in the HA‐based hydrogels proposed here, combined with the bioorthogonal and tunable hydrogel cross‐linking chemistry, led us to investigate neural cell encapsulation and further development of bioinks. We employed the human neuroblastoma cell line SH‐SY5Y for initial evaluation and optimization of the culturing conditions. This cell line has been used extensively as a model system for neurodegenerative disease in 2D and 3D cultures,^[^ ^78^, ^79^, ^80^, ^81^ ^]^ and can also be differentiated into a more expansive and branched neuronal phenotype. SH‐SY5Y was thus considered as a robust option for initial exploration of the hydrogel system for neural cell culture and biofabrication, prior proceeding with the sensitive but more clinically and translationally relevant human iPSC‐derived NSCs.

**Figure 3:**
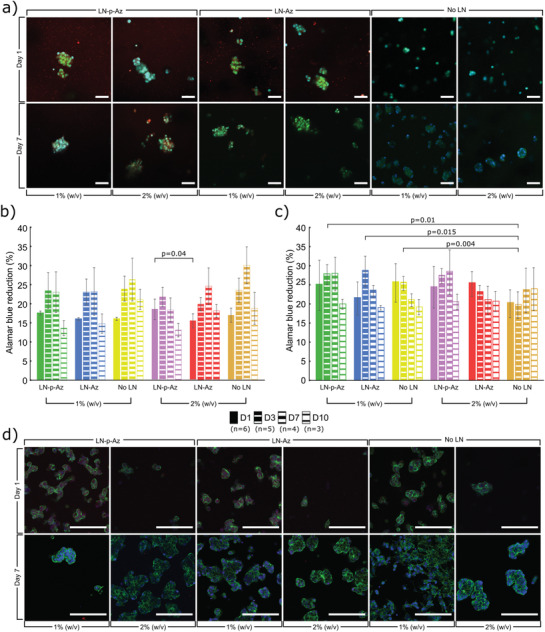
a) Confocal images of undifferentiated SH‐SY5Y cells cultured in 1% and 2% (w/v) HA‐BCN:PEG hydrogels with and without LN‐(p)‐Az, stained for F‐actin (Phalloidin, green) and Hoechst nuclear dye (blue). LN was labeled with Cy3 (red). AB cell viability of b) undifferentiated SH‐SY5Y and c) RA treated differentiated SH‐SY5Y cells, cultured in HA:PEG hydrogels with and without LN‐(p)‐Az, N as indicated on figure legend for each condition, where each data point represents a separate hydrogel. Error bars are standard deviation. Significance was calculated using one‐way ANOVA followed by Tukey's HSD. d) Confocal images of RA treated differentiated SH‐SY5Y cells cultured in 1% and 2% (w/v) HA:PEG hydrogels with and without LN‐(p)‐Az, stained for F‐actin (Phalloidin, green) and Hoechst nuclear dye (blue). Cell seeding density is 2000 cells per µL of hydrogel precursor. Scale bars: 100 µm.

We first cultured undifferentiated SH‐SY5Y cells in the hydrogels with and without LN. Samples imaged at time points of 1‐, 3‐, and 7‐days showed even cell dispersal across all conditions with cells forming small multicellular spheroids within the hydrogels with few truncated processes (Figure S2, Supporting Information), which is characteristic for undifferentiated SH‐SY5Y cells. We used confocal imaging to confirm the spheroid‐like morphology observed (**Figure** **3**a; Figure S3, Supporting Information). The viability of the encapsulated cells was determined using an AB assay, which revealed high metabolic activity for the cells over 10 days, with no or minor differences between the conditions concerning LN‐functionalization hydrogel concentration (Figure 3b). Thus, with or without the added functionalization of LN, the HA:PEG hydrogel system can efficiently sustain the neuroblastoma cells. Proliferation decreased in all conditions after day 7, which we hypothesize is a consequence of an increasing spheroid diameter over time, that might lead to oxygen and nutrient starvation of cells in the core of the spheroids, resulting in necrosis.^[^ ^82^ ^]^ The nonsignificant effects of LN on SH‐SY5Y cell proliferation indicate that cells may adhere to the HA backbone, making any other interactions with LN redundant. The main cell surface receptor for binding to HA is CD44, a family of transmembrane cell surface glycoproteins that plays an important role in cell–cell and cell–matrix interactions and is linked to the tumorigenic properties of neuroblastoma cells. Subpopulations of SH‐SY5Y cells have been shown to express CD44.^[^ ^83^ ^]^ As indicated by immunostaining, the undifferentiated SH‐SY5Y cells express CD44 when cultured in HA:PEG, both in the absence and presence of LN‐p‐Az, providing additional means for cell adhesion to the hydrogels in addition to integrin–LN interactions (Figure S4, Supporting Information).

To induce differentiation of the SH‐SY5Y cells, retinoic acid (RA, 10 µm) was added to the cell culture medium 7 days before encapsulation in the hydrogels. RA has highly potent growth‐inhibiting and cellular differentiation‐promoting properties and triggers differentiation, primarily to a cholinergic neuronal phenotype.^[^ ^84^, ^85^ ^]^ The differentiated cells remained viable in all hydrogels up to 10 days in culture as indicated by the AB assay (Figure 3c). We observed that the cells assembled into spheroids in all conditions. This process can be influenced by both hydrogel stiffness and presence of LN. The inner cell mass in spheroids typically experience a lower rate of proliferation due to the oxygen and nutrient gradient.^[^ ^86^ ^]^ Since LN offer better interactions with the hydrogel scaffold, spheroid formation can be delayed under these conditions, which can explain the higher proliferation at day 3 in the presence of LN. The decrease in proliferation at day 7 for all conditions is consistent with an increasing size of the spheroids. Interestingly, the viability of the differentiated SH‐SY5Y cells rapidly decreased in hydrogels supplemented with nonconjugated LN (Figure S5, Supporting Information). RA differentiation of SH‐SY5Y cells increases the expression of *α*_3_ *β*_1_ integrin heterodimers,^[^ ^87^, ^88^ ^]^ which interact strongly with LN‐521.^[^ ^89^, ^90^ ^]^ Binding of nonconjugated LN‐521 thus likely interferes with cell–hydrogel binding. Loss of cellular attachment to ECM may trigger programmed cell death via anoikis pathways, which is a specific form of apoptosis.^[^ ^91^ ^]^ Confocal images of the differentiated cells showed homogenously distributed cell clusters in all conditions, growing into larger spheroids by day 7 (Figure 3d), similar in size and geometry as SH‐SY5Y cultured hydrogels of collagen or alginate.^[^ ^92^, ^93^ ^]^

### 3.4 lt‐NES Viability during Spontaneous 3D Differentiation

**Figure 4:**
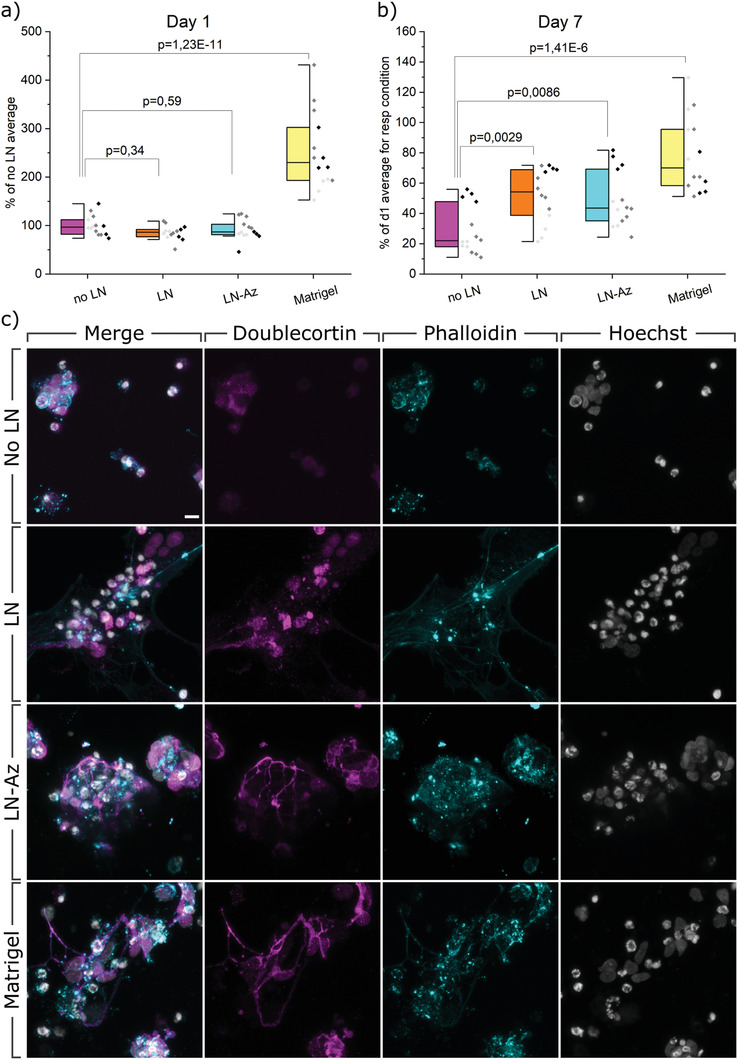
AB viability assay and confocal images of spontaneous 3D differentiation. a) Viability measured with AB after 1 day of differentiation of lt‐NES in respective hydrogels. b) Viability measured with AB after 7 days of differentiation of lt‐NES in respective hydrogels. Data were collected from three individual experiments (indicated with different shades of gray), *N* = 14, where *N* represents one hydrogel replicate. *p*‐values were derived using LMM with all data included. Data are presented both as a box indicating the 25th–75th percentile with a median line and ±1.5 IQR whiskers, and as individual data points. c) Confocal images of spontaneous 3D differentiation in respective hydrogels. lt‐NES are stained with neuronal marker DCX (magenta), cytoskeletal F‐actin marker Phalloidin (cyan), and nuclear stain Hoechst (gray). Scale bar: 10 µm.

Whereas the 3D cultures of neuroblastoma cells represent an important neural disease model, the potential to encapsulate and culture lt‐NES in the HA‐PEG hydrogels offers opportunities also to explore models of healthy tissues, advanced models of genetic disorders, and development of cell‐based therapeutic strategies. Neural progenitor cells and lt‐NES spontaneously differentiate into mixed cultures of high (80–95%) percentage neurons and some glial cells. lt‐NES have been used in several studies both as disease models and as a source to create healthy neurons, both in 2D and 3D.^[^ ^94^, ^95^, ^96^ ^]^ Here, lt‐NES were encapsulated in the HA:PEG hydrogels with the addition of LN, with and without an Az conjugation. To benchmark these defined hydrogels as a matrix for cultivation and differentiation of the sensitive lt‐NES, we used commercially available Matrigel. The 1% HA:PEG hydrogels were used from here on due to the better match with the stiffness of Matrigel. Before seeding the lt‐NES in the hydrogels, we allowed them to spontaneously predifferentiate in 2D cell culture flasks for 5 days, from now on referred to as predifferentiated lt‐NES. At this stage of differentiation, cell proliferation is reduced, and a high degree of cell death can be observed in 2D cultures. Cells that did not take on the differentiation and thus died were discarded and the remaining predifferentiated lt‐NES were encapsulated in the hydrogels, where the cells started exhibiting signs of early neural profile in terms of morphology and gene expression. After 1 day of the subculture in the hydrogels, we measured lt‐NES metabolic activity using AB, demonstrating viable cells in all gel conditions (**Figure** **4**a). Whereas we observe no or only minor differences in viability of the conditions with/without LNs, the viability is about 2–3 times higher in Matrigel. Matrigel is tumor derived, composed by several partially fragmented ECM proteins, and contains traces of growth factors that can give higher cell survival and proliferation of the lt‐NES throughout the first 24 h of culture, as confirmed by brightfield microscopy (Figure S8, Supporting Information). Additionally, we observe that the Matrigel encapsulated cells result in a larger spread of data points in viability compared to the other hydrogel conditions with an interquartile range (IQR) of [193;302], compared to hydrogels without LN [82;112], nonconjugated LN [77;112], and LN‐Az [81;103]. The well‐defined HA:PEG hydrogels thus clearly provide better reproducibility between independent experiments than Matrigel (Figure 4a). After 7 days of subculture in the hydrogels (Figure 4b), a significant difference in viability was observed in hydrogels containing LN. However, in contrast to the differentiated SH‐SY5Y cells, conjugation of the LN does not appear to change the viability of the lt‐NES compared to nonconjugated LN. Similar to what we observe after 1 day of subculture, the viability of the cells in Matrigel is significantly higher and shows a larger spread of the individual data points (indicated as different shades of gray). An analysis of the IQR shows that Matrigel had the largest variability [58;96] compared to the conditions with no LN [35;69], LN [39;69], and LN‐Az [18;48]. We hypothesize that the increase in viability in Matrigel compared to day 1 is partly due to a continuous proliferation throughout the 7 days of subculture, in line with the higher proliferative potential of that material as mentioned above, caused by its various components such as mixed ECM proteins and growth factors traces.

### 3.5 lt‐NES Morphology after 3D Differentiation

More specific assessments of neuronal differentiation require characterization of neuronal markers both on an imaging and mRNA expression analysis level. As seen by immunocytochemistry (Figure 4c), lt‐NES cultured in the HA:PEG hydrogels without LN are generally characterized by singularly distributed cells and smaller clusters of cells. The expression of DCX, an early marker of neuronal differentiation, is mainly limited to the area surrounding the individual nuclei, and occasional neurite outgrowths are found. Phalloidin (F‐actin stain) shows how the soma is rounded up, and little cell spreading or contact with the hydrogel is seen. Singularized nuclei appear brighter and condensed to a higher level compared to clustered nuclei. A larger proportion of loosely clustered single cells is observed when adding LN to the hydrogels, while some smaller clusters are still present. Similar to the no LN condition, DCX expression is limited to an area around the cell nuclei. The cells form small clusters with connected processes, confirmed by F‐actin staining. The F‐actin staining further visualizes cell–cell connections and close interaction between the cells and the hydrogel. In the LN conditions mixed morphologies of nuclei are observed, some are larger and more oval‐shaped, and others are brighter and more condensed close to pycnotic, much like those seen in the condition no LN. In the HA:PEG hydrogels with conjugated LN, Az‐LN, the cell distribution appears similar to the LN condition, with respect to singular cells and small clusters. DCX expression apart from the soma is detectable in neurite outgrowths connecting cells in the clusters. F‐actin staining reveals a somewhat condensed cytoskeletal structure indicating strong cell–cell interactions. Most nuclei appear larger and oval‐shaped when in the loose clusters, indicating that cells are mostly healthy, although some nuclei are brighter and more condensed. In the Matrigel condition, cells are growing both singularized and in less tightly packed clusters, and we observe that the cells send out longer processes between each other. The expression of DCX extends from the soma to the outer processes The spreading and outreach of the cells are confirmed by the cytoskeletal constructs seen in Phalloidin, suggesting that the cells in a similar way to the LN condition can attach to their microenvironment. We further confirm neuronal fate specification of the differentiated lt‐NES by expression of the later neuronal marker TUBB3 with immunocytochemistry (Figure S7, Supporting Information).

Depending on the size and format of the 3D‐hydrogel being stained, lengthy immunostaining protocols are needed, compared to 2D cultures, to provide enough diffusion time for antibodies to penetrate the gel and bind to the cells. With Matrigel, we found it difficult to avoid unspecific binding and high background noise even with repeated and longer washing steps. Issues like these can prove disruptive to imaging, especially image analysis, since larger clusters of background noise can be easily similar in size to thin neurites or other structures of interest in NSC cultures. Notably, we did not experience background noise issues and unspecific binding with HA:PEG‐based hydrogels, which, on top of its defined formula, gives HA:PEG one more advantage over Matrigel.

### 3.6 mRNA Expression Analysis

**Figure 5:**
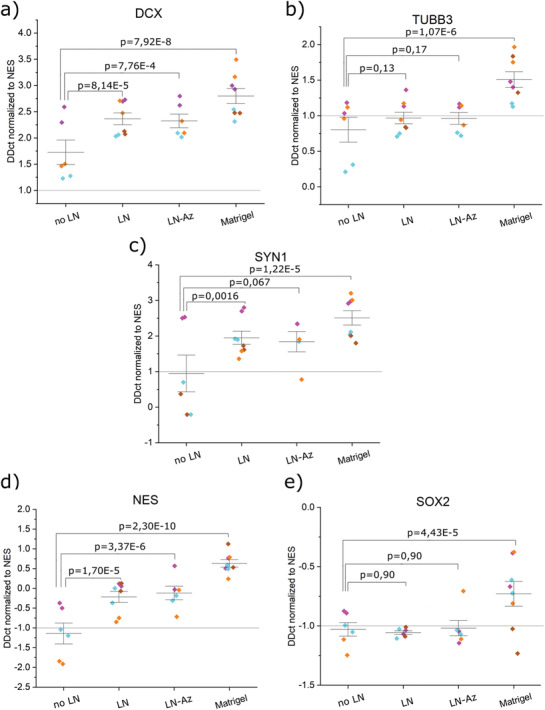
mRNA analysis profile of spontaneous 3D differentiation of lt‐NES in different hydrogel conditions. a) DCX, b) TUBB3, c) SYN1, d) NES, and e) SOX2. All samples contain the housekeeping gene GAPDH. *p*‐values were derived with LMM, and data were normalized to the hydrogel condition no LN. Data is collected as duplicates from three or four independent experiments (indicated by different colors), with the whiskers indicating ±1 SE around the mean (bar).

After 5 days of spontaneous predifferentiation in conventional cell culture flasks and 7 days of continued spontaneous differentiation in 3D‐hydrogels, we extracted RNA in the typical range of 5–20 ng µL^−1^ from 50 µL hydrogels. We observe significant changes in gene expression in most of the analyzed genes, both between some hydrogel conditions (**Figure** **5**) and compared to the undifferentiated state of lt‐NES (Figure S6, Supporting Information). The neuronal marker DCX (Figure 5a) is upregulated (1.75–2.75 times) in all hydrogel conditions and more so when LN was added to the hydrogels. However, we see no significant effect of LN conjugation. Cells differentiated in Matrigel showed the highest upregulation of DCX, supported by our imaging findings (Figure 4c). When compared to the undifferentiated state of lt‐NES (Figure S6, Supporting Information), we observe that all conditions had significant upregulation of DCX, which is in line with previous studies where neuroepithelial stem cells show expression of DCX after 7 days of neural differentiation.^[^ ^97^ ^]^ The later neuronal marker TUBB3 also shows statistically significant upregulation compared to undifferentiated lt‐NES (Figure S6, Supporting Information), though based on its magnitude (1–1.5 times; Matrigel again showing largest change) likely to have only minor biological impact. When comparing expression between the different hydrogel conditions (Figure 5b), however, we do not see any significant up or downregulation when adding LN or LN‐az. We observe a statistically significant change in Matrigel, where (similar to the lt‐NES comparison) TUBB3 is upregulated 1.25–2 times, indicating minor changes biologically. As a measure of cell attachment and interaction with the hydrogels, including synaptogenesis, we investigate the expression of SYN1. We observed an upregulation in all conditions, with the addition of LN making a marginal (≈1‐fold) but statistically significant difference in upregulation independent of Az conjugation (Figure 5c). The highest upregulation we see in Matrigel (1.75–3.25 times).

As another measure of neuronal differentiation, we include two stem cell markers, NES and SOX2, expecting that these two genes should be downregulated in the case of successful neuronal differentiation. SOX2 has a critical role in maintaining pluripotency and directing pluripotent stem cells to neural progenitors.^[^ ^98^ ^]^ A previous study using human neuroepithelial stem cells to create human midbrain organoids reported a change from 35% to 18% positive SOX2 cells when comparing expression at 27 days and 61 days of differentiation, respectively.^[^ ^99^ ^]^ We have seen in prior work that spontaneous differentiation of lt‐NES in 2D‐cultures result in downregulation of SOX2 after 28 days.^[^ ^100^ ^]^ Our results show significant downregulation of NES in the HA:PEG hydrogels without LN, but not when any type of LN was added. Compared to the no LN condition, we observe a marginal upregulation of NES in the Matrigel condition, indicating that the stem cell state would be more preserved for the cells cultured in Matrigel. As for SOX2, we see a clear downregulation in all hydrogel conditions, with no difference if any kind of LN is added. The data have high variability in the Matrigel condition, and no significant downregulation can be concluded in terms of change in DDct values. Similar to our observations in SOX2 regulation, such high variability and lack of downregulation of NES, as we see in the other hydrogel conditions, gives Matrigel a disadvantage as a matrix for neuronal differentiation compared to the HA:PEG‐based hydrogels.

For all the genes, we observe the largest variation in data points from the no LN condition, which was also the condition that, in general, had the lowest mRNA yield. In summary, these hydrogels support culture of the sensitive lt‐NES, however they do not appear to strongly enhance neuronal differentiation with network formation or synaptogenesis for short time differentiations.

### 3.7 Ejection of Predifferentiated lt‐NES in HA:PEG, Collagen, and Media

**Figure 6:**
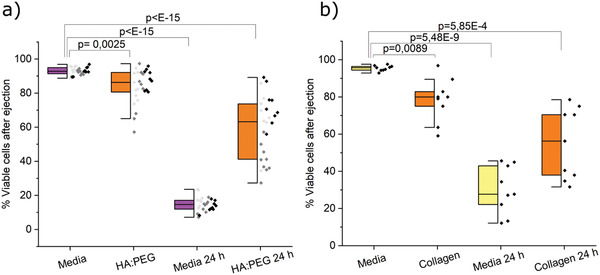
a) Survival of ejected predifferentiated lt‐NES in HA:PEG and cell media immediately after ejection and after 24 h measured with Live/Dead assay. Data were collected from three individual experiments (indicated with different shades of gray), *N* = 27, where *N* represents one replicate of ejected cells. Outliers were removed by a Grubbs Test, and *p*‐values were derived using LMM in Origin Pro. Data are presented both as a box indicating the 25th–75th percentile with a median line and ±1.5 IQR whiskers, and as individual data points. b) Survival of ejected predifferentiated lt‐NES in collagen and cell media. Data were collected from one individual experiment, *N* = 9, where N represents one ejected replicate. No data were excluded. *p*‐values were derived using LMM in Origin Pro.

In addition to providing a suitable matrix for 3D culture of sensitive neural cell models, the defined composition combined with the bioorthogonal cross‐linking chemistry of the HA:PEG hydrogel can facilitate implementation of cell‐based regenerative therapies.^[^ ^101^, ^102^, ^103^, ^104^ ^]^ Syringe‐based cell transplantation exposes the cells to significant shear forces that may mechanically disrupt the cells and substantially reduce cell viability. In many transplantation studies, PBS or cell media is used as a vehicle to carry the cells. However, shear‐thinning hydrogels have been demonstrated to provide a protective effect during the injection.^[^ ^105^ ^]^ To investigate the ability of the HA:PEG hydrogels to protect cells experiencing sheer force when ejected through a syringe needle, we compared the viability of predifferentiated lt‐NES in an HA:PEG matrix to cell media and a collagen gel both in an acute state and after 24 h by assessing the amount of live and dead cells. Collagen is one of the most widely employed biomolecules for generating hydrogels, notably with gelling and handling characteristics more conducive to injection‐ or extrusion‐based approaches than Matrigel (and more similar to HA:PEG).^[^ ^106^ ^]^ Thus, it serves as a convenient baseline here to optimize our ejection platform, before turning to the material of interest, HA:PEG hydrogels, that in addition to forming a chemically cross‐linked hydrogel after ejection would be of more clinical relevance for future applications. At the acute state, we observed reduced viability of the HA:PEG ejected cells compared to those ejected in cell media (**Figure** **6**a). The same effect was seen when comparing cells ejected in a collagen matrix compared to cell media (Figure 6b). However, after 24 h, cells ejected in the HA:PEG matrix showed higher viability than cells ejected in cell media, suggesting that the hydrogel provides a protective environment during and after syringe ejection.

### 3.8 3D Bioprinting

**Figure 7:**
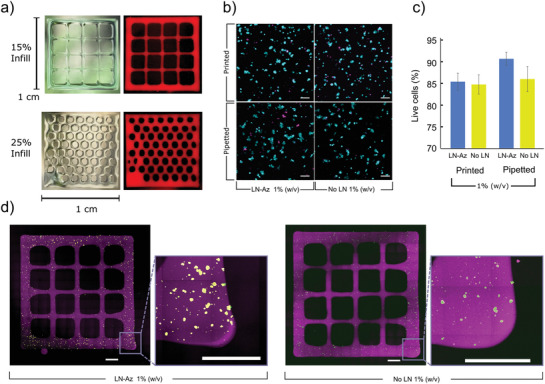
a) 3D bioprinted structures based on the HA:PEG‐LN hydrogels at a concentration of 1% (w/v). Hydrogels (red) were dyed with Cy5 and illuminated using a white light source. b) Live (cyan)/Dead (magenta) staining of SH‐SY5Y cells 24 h after bioprinting. c) SH‐SY5Y cell viability 24 h after bioprinting or pipetting when encapsulated in either HA:PEG (without LN) and HA:PEG‐LN hydrogels at a concentration of 1% (w/v), *N* = 4 bioprinted/pipetted hydrogels were examined for each condition, error bars are standard deviation, statistics: one‐way ANOVA with Tukey's HSD, n.s. = not significant (*p* > 0.05). d) SH‐SY5Y cell bioprinted into grid structures (purple) of Cy5‐labeled HA:PEG‐LN and HA:PEG, respectively, at a hydrogel concentration of 1% (w/v) and imaged using tiled confocal microscopy 24 h after bioprinting. Encapsulated SH‐SY5Y were stained using Live (cyan)/Dead (magenta) staining. Inset square indicates a magnified portion. Scale bars are 1000 µm.

The protective effect of the HA:PEG hydrogels on cells during syringe ejection is also a highly attractive feature for 3D bioprinting applications. To assess the printability of the hydrogels, hydrogel lattices (1 × 1 cm) were fabricated using a Cellink BioX bioprinter (**Figure** **7**a; Figure S9, Supporting Information). Since the gelation kinetic for this hydrogel system is highly temperature dependent and proceeds significantly slower at room temperature (RT) than at 37 °C, we incubated the bioinks at RT for about 10 min prior printing to partially cross‐link the hydrogels. This process resulted in bioinks with a viscosity that was suitable for bioprinting. In addition to enabling printing of features with dimensions <400 µm, the hydrogels supported high viability of the bioprinted SH‐SY5Y cells (Figure 7b,c). Similar to syringe‐based cell ejection, 3D bioprinting exposes cells to substantial shear forces that can be detrimental for cell viability due to the rapid change in fluid velocity when the cell suspension is forced from the syringe into the much smaller diameter needle, resulting in cell rupture.^[^ ^107^, ^108^ ^]^ By encapsulating the cells in the HA:PEG hydrogels matrix, the cells were protected from the lethal shear forces during bioprinting. SH‐SY5Y cells encapsulated in LN‐functionalized HA:PEG showed high (>85%) viability 24 h after printing, similar to cells carefully extruded through a pipette (Figure 7c) and on par with carefully optimized alginate‐based bioinks.^[^ ^109^ ^]^ Moreover, the bioprinted SH‐SY5Y cells showed a similar morphology and distribution in the 3D bioprinted structures as when cultured in the casted hydrogels (Figure 7b), indicating the potential of this hydrogel system for 3D bioprinting of neural disease models. Interestingly, 3D bioprinting of the SH‐SY5Y cells in HA:PEG hydrogels functionalized with LN resulted in a higher density of spheroids than hydrogels without LN (Figure 7d,e).

**Figure 8:**
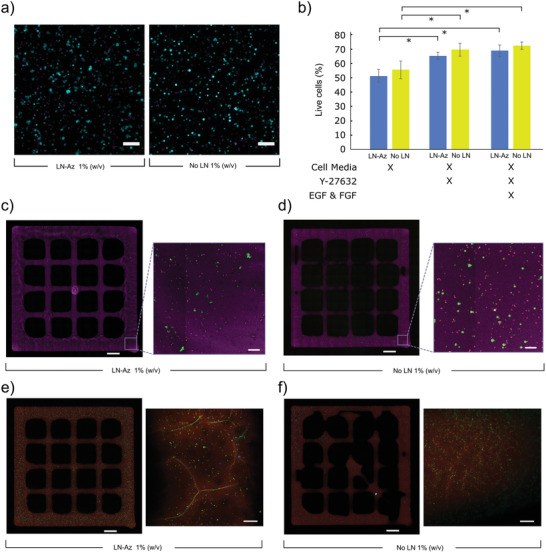
Bioprinted predifferentiated lt‐NES cells in HA:PEG hydrogel. a) Live (cyan)/Dead (magenta) staining of lt‐NES cells 24 h after bioprinting with and without LN. b) Viability of cells in HA:PEG hydrogels with and without LN. Cell media is lt‐NES differentiation media. Cell count includes samples whereby the media was supplemented with Y‐27632 (apoptosis inhibitor) and EGF and FGF. *N* = 5, representing individual wells. Error bars represent standard deviation. Statistical analysis: ANOVA with Tukey's HSD, * = *p* < 0.05. c) Bioprinted predifferentiated lt‐NES seeded grids of HA:PEG‐LN and d) HA:PEG stained using Live (red)/Dead (green) staining with the hydrogel labeled with Cy5‐Az (magenta). Inset square indicates a magnified part of the printed grids. e) Bioprinted lt‐NES seeded grids of HA:PEG‐LN and f) HA:PEG incubated for 10 days and stained for TUBB3 (green), Hoechst (blue) and hydrogel labeled with Cy5‐Az (red). Scale bars: 1000 µm on full size grids and 100 µm on all other images.

After successful optimization of the printing conditions using the SH‐SY5Y cells, we proceeded with exploring possibilities to bioprint the predifferentiated lt‐NES cells. The cells were encapsulated in HA:PEG hydrogels with and without LN and printed using the same conditions as for the SH‐SY5Y cells. Viability was assessed 24 h postprinting using Live/Dead staining (**Figure** **8**a). Cell viabilities of about 50–55% were obtained with no difference between the two conditions (Figure 8b). We noted a significant increase in cell viability after printing when supplementing the medium with an apoptosis inhibitor (Y‐27632), resulting in about 70% viable cells 24 h after printing. Supplementing the cells with epithelial growth factor (EGF) and fibroblast growth factor (FGF), in addition to Y‐27632 during and after printing, did not result in any additional increase in cell viability. The lt‐NES were well distributed in the bioprinted structures as indicated by the Live/Dead stain (Figure 8c,d). The bioprinted predifferentiated lt‐NES were cultured for an additional 9 days (10 days in total) prior staining for TUBB3 to observe neurite outgrowth, cell morphology, and maturity (Figure 8e,f). Both conditions demonstrated expression of TUBB3, with more pronounced processes in hydrogels containing LN (Figure S10, Supporting Information).

In summary, we presented a tunable and modular HA‐based LN‐521 functionalized hydrogel that can effectively retain LN over 7 days, showing a successful conjugation of the LN to the hydrogel backbone. We show that the hydrogel supports proliferation of the widely used neural cell model SH‐SY5Y in both an undifferentiated and differentiated state. The SH‐SY5Y showed high viability after 10 days of subculture in both 1% and 2% hydrogels and appeared to grow in clusters according to F‐actin staining and immunostaining of the HA‐receptor CD44. Choosing the softer more in vivo like 1% hydrogels, we further demonstrated that more sensitive and advanced cell model lt‐NES successfully can be spontaneously differentiated to neuronal fates and develop processes. According to viability assays, LN does not support the cells’ immediate (24 h) survival but does change the viability on a week‐long timescale. Our mRNA expression analysis suggests a significant but biologically limited upregulation in neuronal markers DCX, TUBB3, and SYN1 with the addition of LN to the hydrogels. Our data also suggest that stem cell marker SOX2 is marginally downregulated, whereas we see no significant difference in the expression of the stem cell marker NES with the addition of LN. We proved the possibility of ejecting predifferentiated lt‐NES through a 27G syringe and that adding HA:PEG as an ejection matrix will protect the cells by higher survival after 24 h, compared to cells ejected in cell media. This protective effect of the hydrogel matrix could not be measured through viability at an immediate stage. We furthermore successfully bioprinted SH‐SY5Y cells encapsulated in LN‐functionalized HA:PEG with >85% survival after 24 h, We also observed that the bioprinted cells maintained the same morphology as when cultured in 3D gels, and surprisingly we found that conjugating LN in the hydrogels promoted the formation of spheroids to a larger extent than without the added LN after bioprinting. Finally, we demonstrate successful bioprinting of predifferentiated lt‐NES, that retained high viabilities and expressed TUBB3 10 days after printing with extensive processes in LN containing hydrogels.

## 4 Conclusions

A defined, bioprintable, and tunable hydrogel system that allow for controlled covalent conjugation of the full‐size essential ECM molecule LN was developed. The hydrogel system enabled 3D culture of both undifferentiated and differentiated neuroblastoma cells. The hydrogels were also compatible with sensitive lt‐NES and offer higher reproducibility and simplifies imaging, compared to conventional biologically derived hydrogel systems. Possibilities to process the materials and protect cells during syringe ejection and bioprinting, in combination with being reproducible and completely xeno‐free, can further facilitate biofabrication and development of more advanced neuronal tissue and disease models and facilitate clinical translation of neuronal cell therapies.

## Conflict of Interest

The authors declare no conflict of interest.

## Supporting information

Supporting Information

## Data Availability

Research data are not shared.
